# DNA-Bound Platinum Is the Major Determinant of Cisplatin Sensitivity in Head and Neck Squamous Carcinoma Cells

**DOI:** 10.1371/journal.pone.0061555

**Published:** 2013-04-17

**Authors:** Sanne R. Martens-de Kemp, Simone U. Dalm, Fiona M. J. Wijnolts, Arjen Brink, Richard J. Honeywell, Godefridus J. Peters, Boudewijn J. M. Braakhuis, Ruud H. Brakenhoff

**Affiliations:** 1 Department of Otolaryngology/Head-Neck Surgery, VU University Medical Center, Amsterdam, The Netherlands; 2 Department of Medical Oncology, VU University Medical Center, Amsterdam, The Netherlands; Virginia Commonwealth University, United States of America

## Abstract

**Purpose:**

The combination of systemic cisplatin with local and regional radiotherapy as primary treatment of head and neck squamous cell carcinoma (HNSCC) leads to cure in approximately half of the patients. The addition of cisplatin has significant effects on outcome, but despite extensive research the mechanism underlying cisplatin response is still not well understood.

**Methods:**

We examined 19 HNSCC cell lines with variable cisplatin sensitivity. We determined the *TP53* mutational status of each cell line and investigated the expression levels of 11 potentially relevant genes by quantitative real-time PCR. In addition, we measured cisplatin accumulation and retention, as well as the level of platinum-DNA adducts.

**Results:**

We found that the IC_50_ value was significantly correlated with the platinum-DNA adduct levels that accumulated during four hours of cisplatin incubation (p = 0.002). We could not find a significant correlation between cisplatin sensitivity and any of the other parameters tested, including the expression levels of established cisplatin influx and efflux transporters. Furthermore, adduct accumulation did not correlate with mRNA expression of the investigated influx pumps (*CTR1* and *OCT3*) nor with that of the examined DNA repair genes (*ATR*, *ATM*, *BRCA1*, *BRCA2* and *ERCC1*).

**Conclusion:**

Our findings suggest that the cisplatin-DNA adduct level is the most important determinant of cisplatin sensitivity in HNSCC cells. Imaging with radio-labeled cisplatin might have major associations with outcome.

## Introduction

Head and neck squamous cell carcinomas (HNSCCs) arise in the mucosal linings of the upper aerodigestive tract. Early stage tumors are treated with surgery or radiotherapy and these treatment modalities are combined to treat more advanced tumors. Since recent years chemoradiation protocols are increasingly applied to treat locally advanced carcinomas, consisting of systemic application of cisplatin (cis-diammine-dichloroplatinum II) combined with locoregional radiotherapy. The addition of cisplatin causes a significant increase in survival rates [1] and has become standard treatment. Despite these promising results, only 50% of patients are cured by chemoradiation. The tumors that do not respond well might better have been treated by surgery combined with postoperative radiotherapy. Therefore, biomarkers for personalized therapy are urgently awaited, and these might be found among factors that determine cisplatin and radiation response. It is conceivable that cells acquire resistance to cisplatin during treatment, but also intrinsic resistance of chemo-naive cells might be important. It is necessary to fully understand the mechanism of cisplatin response in order to find ways to personalize treatment, to sensitize tumor cells to this anticancer drug or to protect normal cells.

Several mechanisms of cisplatin resistance have been described previously (reviewed in [2], [3]), and it has become clear that the cellular processing of platinum provides important insights into the molecular mechanisms involved in cisplatin response. With the observation that the kinetics of uptake and efflux of cisplatin appears to follow a parallel pattern with those of copper [4], [5], *CTR1* was identified as an important cisplatin influx transporter [4], [6]–[9]. *ATP7A* [4], [10], *ATP7B* [4], [11]–[13] as well as members of the organic cation transporter (OCT) family [14] are reported to play an important role in cisplatin efflux. Altered expression of these proteins has also been correlated to poor outcome in several tumor types, including HNSCC [15], [16].

Once inside the cell, cisplatin looses the chloride groups as a result of the low cellular chloride concentration, a process called aquation. This hydrolyzed form is more reactive to cellular targets of which the nuclear DNA is biologically most relevant. Cisplatin exerts its cytotoxic effect by the formation of intrastrand and interstrand DNA adducts, resulting in cell cycle arrest and eventually cell death. Cells may survive the toxic DNA damage by increased DNA repair activities that lead to the removal of cisplatin adducts. Nucleotide excision repair (NER) is one of the pathways known to remove cisplatin lesions from the DNA [17]. *ERCC1* is one of the key factors in the NER system, and the expression of this endonuclease was identified as a prognostic marker in several types of cancer, including HNSCC [18], [19]. Unrepaired cisplatin-DNA adducts eventually result in stalled replication forks during the process of DNA replication in the S-phase of the cell cycle. The cell is then forced into an S-phase arrest to allow time for DNA repair, for example via translesion synthesis or homologous recombination. Members of pathways involved in these processes are described to be extremely important in the process of crosslink repair, and deficiencies in two important players, *BRCA1* and *BRCA2*, sensitize cells to cisplatin treatment [20], [21]. The Fanconi anemia (FA) pathway also proved to be essential in the repair of cisplatin induced DNA damage, since patients with defects in one of the 15 known FA genes show cellular hypersensitivity to DNA cross-linking agents, including cisplatin [22]. Also, ATM and ATR, the proteins that are responsible for the detection of the initial DNA damage and for the initiation of the actual DNA repair process, are likely essential to establish a good DNA repair response.

Furthermore, the *TP53* gene, an important player in cellular homeostasis, has also been associated with cisplatin response. It was demonstrated that mutated and inactivated *TP53* influences cisplatin sensitivity [23]–[25]. This gene is of special interest concerning its frequently mutated status in human tumors, including HNSCC [26]–[28]. In HNSCC cells the G1 and G2 cell cycle checkpoints can be impaired by *TP53* mutations, loss of *CDKN2A* (p16^INK4a^ *)* or gain of *CCND1* (Cyclin D1). This might result in the entrance of tumor cells into S-phase despite the presence of cisplatin adducts, leading to cell death.

Despite the importance of the aforementioned genes in the pharmacodynamics and anti-tumor effect of cisplatin, the actual mechanism of cisplatin response in HNSCC is still not understood. Many genes seem to be important in this response, but their relevance may vary from one tumor model to another and affect cisplatin activity at different levels (influx, efflux, metabolism or DNA repair). In the present study we characterized cisplatin sensitivity and the cellular characteristics of 19 HNSCC cell lines, the largest panel tested as far as we know. We investigated the expression levels of several genes that are implicated in cisplatin toxicity and measured cisplatin accumulation and retention rates as well as cisplatin-DNA adduct levels in these cell lines. Finally, we investigated potential correlations between the different parameters studied and the cellular sensitivity to cisplatin.

## Materials and Methods

### Cell Lines and Chemicals

HNSCC cell lines used were all cultured in Dulbecco's modified Eagle's medium (DMEM), 5% fetal calf serum (FCS) and 2 mM L-glutamine, in a humidified atmosphere of 5% CO_2_ at 37°C. Cell lines of sporadic tumors were established as described previously [29] and are indicated as VU-SCC-040 (formerly known as 92VU040), VU-SCC-094 (formerly 93VU094), VU-SCC-096 (formerly 93VU096), VU-SCC-120 (formerly 93VU120), VU-SCC-147 (formerly 93VU147) and VU-SCC-OE. Three HNSCC cell lines were established from patients carrying a mutation in one of the Fanconi anemia genes [30]; VU-SCC-1131 (formerly VU1131, with mutations in *FANCC*), VU-SCC-1365 (formerly VU1365, with mutations in *FANCA*) and OHSU-0974 (with mutations in *FANCA*). Cell line VU-SCC-9917 was newly established from a sporadic oral cavity tumor, after written informed consent was obtained. The studies involving clinical specimens were according to the Dutch legislations on research with human material. This study was approved by the Institutional Review Board (Medisch Ethische Toetsingscommissie VUmc).

Cell lines UM-SCC-6, UM-SCC-11B, UM-SCC-14A, UM-SCC-14B, UM-SCC-14C, UM-SCC-22A, UM-SCC-22B and UM-SCC-38 were obtained from Dr. T. Carey (University of Michigan) [31]. The commercially available cell line FaDu was obtained from the American Type Culture Collection. All cell lines have been genetically characterized by microsatellite markers to allow authentication. All cell lines were tested for human papillomavirus (HPV) and only cell line VU-SCC-147 is HPV-positive [32].

Cisplatin was obtained from Teva Pharmachemie (Haarlem, The Netherlands) in a concentration of 1 mg/ml.

### IC_50_ Analysis

Cells were plated in 96 well plates in concentrations that allow the cells to grow till 70% confluence after 5 days (ranging from 1,000–5,000 per well, optimized per cell line). After 24 hours, cells were treated with 18 different concentrations of cisplatin (0–666 µM). Plates were incubated for 72 hours at 37°C/5% CO_2_. Cell viability was determined by 2 hours incubation with 20 µl of a 1∶1 dilution of CellTiter-Blue (Promega, Leiden, The Netherlands) with culture medium. Fluorescence was measured using an Infinite 200 microplate reader (Tecan, Männedorf, Switzerland).

### *TP53* Mutation Analysis

Direct dideoxynucleotide sequencing of the evolutionary conserved regions of *TP53* (exons 5–9) was performed on all 19 HNSCC cell lines as described previously [28]. In case mutations were not found in these exons, the remaining coding exons (exons 2–4, 10 and 11) were sequenced in addition. Scored mutations were classified according to criteria described by Lindenbergh-van der Plas et al. [28], dividing the tested cell lines into two distinctive groups. Mutations were assigned “truncating” in case of a deletion or insertion. Mutations introducing a stop codon or a splice site change resulting in a frame shift were also called “truncating”. All other mutations leading to an amino acid change were considered “missense”.

### Quantitative Real-time PCR

Cell pellets were made from cultures grown to 70% confluence. RNA was extracted using TRIzol (Invitrogen, Bleiswijk, The Netherlands), and quality control tests were performed using the Nanodrop (Thermo Fisher Scientific, Landsmeer, The Netherlands). Complementary DNA was synthesized from 250 ng of RNA template using AMV reverse transcriptase (Promega, Leiden, The Netherlands) and a custom designed reverse primer specific for the gene of interest. Amplification of the cDNA was performed on the ABI/Prism 7500 Sequence Detector System (Taqman-PCR, Applied Biosystems, Nieuwerkerk aan den IJssel, The Netherlands) with 1× Power SYBR Green PCR Master Mix (Applied Biosystems, Nieuwerkerk aan den IJssel, The Netherlands) and custom designed primers for each gene of interest. The primer sequences are displayed in Table S1. For each sample the cycle number at which the amount of amplified target crossed a pre-determined threshold (the Ct-value) was determined. To correct for differences in RNA input and quality, beta-glucuronidase (*GUSB*) was used as a housekeeping gene [33] for each RNA sample. The mRNA expression was calculated relative to that of *GUSB* using the Delta/Delta Ct method [34].

### Cisplatin Accumulation

Cells were seeded in triplicate in flasks. When 70% confluence was reached, 2 flasks were incubated with 75 µM cisplatin, and the medium of the third flask was refreshed. After four hours, the cells of one cisplatin-incubated flask (the accumulation flask) and the control flask were harvested. The other cisplatin-incubated flask was washed twice with PBS and incubated with fresh medium for another 3 hours, in order to analyze retention of the platinum compound. After counting, cells were resuspended in 0.5 ml 2 M NaOH for overnight incubation at 55°C. Subsequently, 1 ml 1 M HCl was added. Platinum was measured using flameless atomic absorption spectrometry (FAAS) using a Perkin Elmer AS-800 with a THGA graphite furnace (Perkin Elmer,LLC, Norfalk, USA). The instrument response was calibrated across the range 0.2 to 3.0 µM for the whole cell lysate determinations and from 50 to 250 pmol per extract for the DNA adduct determinations (the DNA isolation procedure is specified below). With the graphite furnace 20 µl of individual samples was dried at 110–130°C, ashing was performed at 1300°C and atomization at 2200°C with a read time of 5 seconds.

### Cisplatin-DNA Adducts

Cells were treated as described above for the accumulation and retention experiments. Cells were harvested in culture medium, centrifuged and cell pellets were stored at −20°C until further use. The QiaAmp DNA mini kit (Qiagen, Venlo, The Netherlands) was used to extract genomic DNA from the cell pellets, and the quality was controlled by OD260/280 nm analysis on a Nanodrop (Thermo Fisher Scientific, Landsmeer, The Netherlands). Resulting DNA samples were analyzed for cisplatin content using FAAS as described above.

## Results

### Growth Inhibition

**Figure 1:**
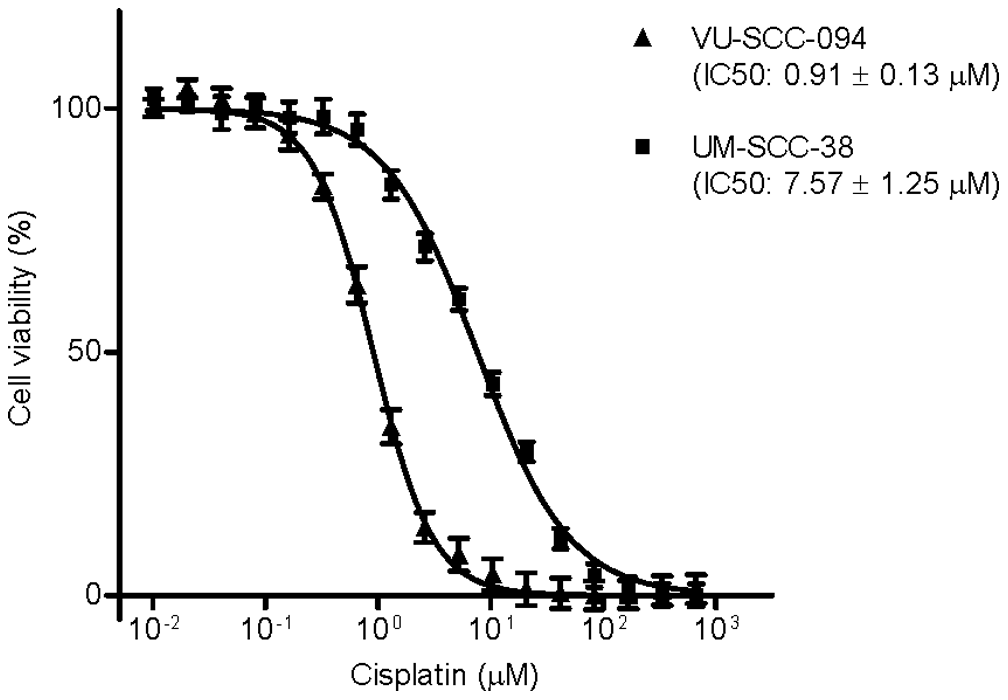
The half-maximal inhibitory concentration of cisplatin. Nineteen HNSCC cell lines were continuously treated with 18 different concentrations of cisplatin during 72 h. Cell viability was determined and plotted against the cisplatin concentration. Cell lines VU-SCC-094 and UM-SCC-38 are presented as examples of a relatively sensitive and a relatively resistant cell line, respectively. Measurements were performed in at least three independent triplicate experiments. Error bars represent the standard error of the mean.

Nineteen head and neck squamous cell carcinoma (HNSCC) cell lines were subjected to treatment with 18 different concentrations of cisplatin, ranging from 0.01 to 666 µM. Examples of resulting curves are shown in Fig. 1. The half-maximal inhibitory concentrations (IC_50_) and the tumor characteristics of the cell lines are displayed in Table S2. Cell lines VU-SCC-1131 and VU-SCC-1365 were among the most sensitive cell lines with IC_50_ values of 0.15 µM and 0.80 µM, respectively. This was not unexpected as both these cell lines were established from patients carrying a mutation in one of the Fanconi anemia (FA) genes. On the other hand, cell line OHSU-0974, also established from a FA patient, did not show an extremely sensitive phenotype (IC_50_ value 2.99 µM). Cell line UM-SCC-38 showed the highest IC_50_ value with 7.57 µM.

There was no significant correlation between the stage of the tumor from which the cell lines were derived and the IC_50_ value (p = 0.297, Spearman’s rho = −0.289). Cell lines UM-SCC-14B, UM-SCC-14C and UM-SCC-22B were left out of this analysis since these were not established from independent primary tumors. When we grouped the cell lines according to their tumor stage (early stage tumors (T1–T2) or late stage (T3–T4)), a significant correlation with cisplatin sensitivity was not found either (p = 0.614, Spearman’s rho = −0.142).

### *TP53* Mutation Status

We investigated the *TP53* mutation status in all 19 cell lines (Table S2). Two cell lines (UM-SCC-6 and VU-SCC-040) harbored wild-type *TP53*, and the other 17 cell lines (88%) showed mutations in the *TP53* gene. Nine cell lines (UM-SCC-14A, UM-SCC-14B, UM-SCC-14C, UM-SCC-22A, UM-SCC-22B, VU-SCC-094, VU-SCC-9917, VU-SCC-OE and OHSU-0974) had truncating *TP53* mutations according to the classification described by Lindenbergh-van der Plas et al. [28]. These mutations included one deletion, two frameshift mutations, 2 splice site mutations and four nonsense mutations that resulted in a stop codon. Cell lines UM-SCC-14A, UM-SCC-14B, UM-SCC-14C, UM-SCC-22A and UM-SCC-22B each harbor two *TP53* mutations; one missense and one truncating mutation. For further analyses, we only counted the truncating mutation since this mutation has most impact on *TP53* functioning. The other eight (47%) cell lines showed one or more missense *TP53* mutations. The *TP53* mutation status (wild type, truncating or missense) did not significantly correlate to cisplatin sensitivity (p = 0.128, Spearman’s rho = 0.600), even when UM-SCC-14B, UM-SCC-14C and UM-SCC-22B were eliminated from the analysis (p = 0.611, Spearman’s rho = 0.138).

### Expression of Genes Related to Cisplatin Sensitivity

**Figure 2:**
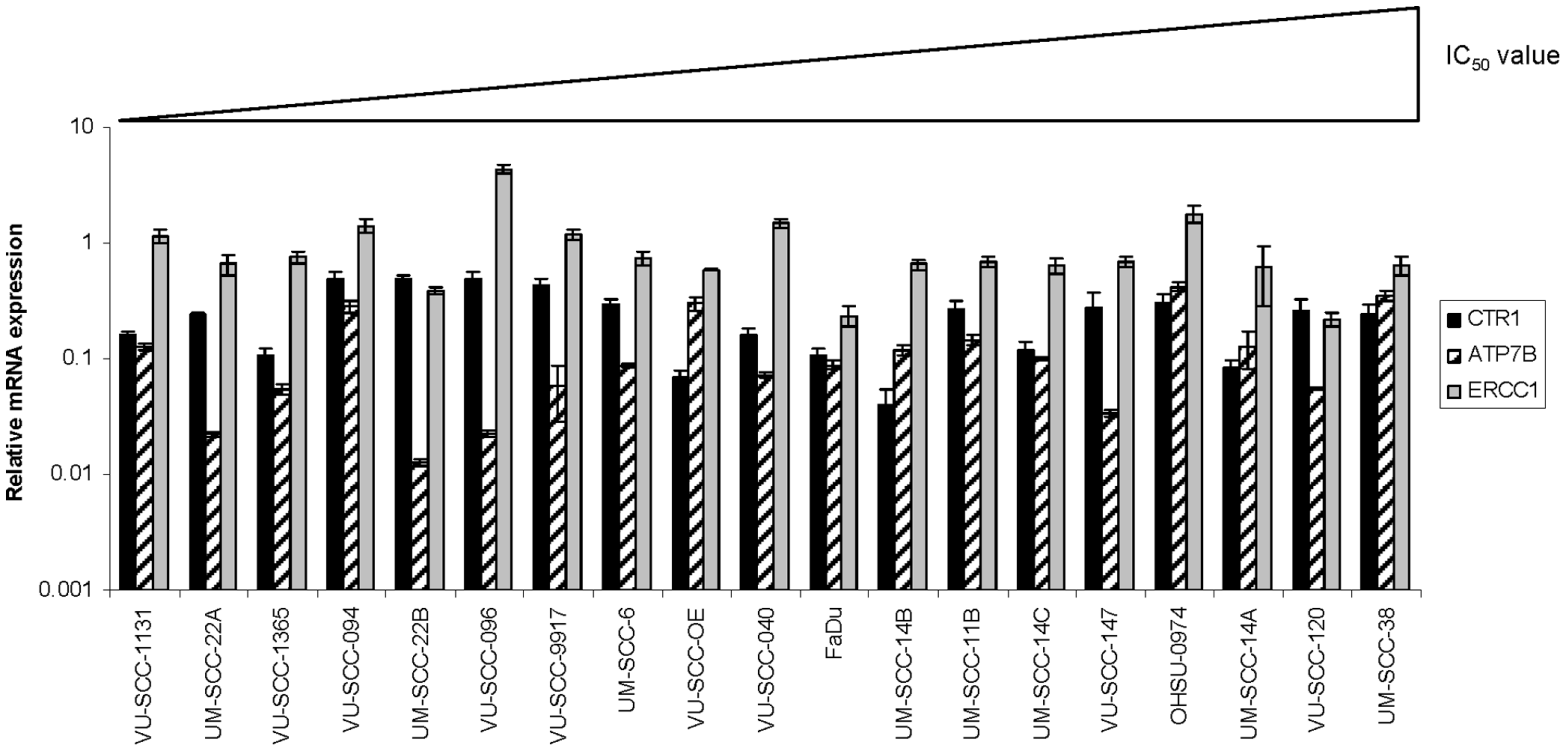
Relative mRNA expression levels of *CTR1*, *ATP7B* and *ERCC*. The mRNA expression level of eleven genes were determined in our HNSCC cell line panel. Genes were selected on basis of their involvement in cisplatin toxicity. CTR1 is believed to mediate cisplatin influx, ATP7B is a known cisplatin efflux pump and ERCC1 is a DNA repair protein. Results are medians of triplicate experiments and are presented relative to the expression of the beta-glucuronidase (*GUSB*) housekeeping gene. Error bars represent standard deviations.

The toxic effect of cisplatin may be dependent on the transport of the compound into the cell, the activation of platinum inside the cell and also the efflux out of the cell (reviewed in [2]). Even when the drug is already bound to the DNA, the toxic effect can be abolished by removal of the formed crosslink. Several genes have been described to mediate cisplatin influx (*CTR1*, *OCT1*, *OCT2* and *OCT3*), efflux (*ATP7A*, *ATP7B*) or are involved in DNA crosslink repair (*ATR*, *ATM*, *BRCA1*, *BRCA2* and *ERCC1*). We used quantitative real-time PCR to investigate the expression of these genes in our cell line panel (Table S3). The expression patterns of *CTR1*, *ATP7B* and *ERCC1* are presented in Fig. 2. The expression of both *OCT1* and *OCT2* could often not be determined due to very low mRNA expression levels. Therefore, these genes were eliminated from further analyses. The mRNA expression levels of the remaining nine genes were correlated to each other as well as to the IC_50_ values, the clinical characteristics of the cell lines and the *TP53* mutation status. Unfortunately, we could not find a significant correlation between the IC_50_ value for cisplatin and the expression of any of the genes tested. Even when we excluded the cell lines with a known mutation in one of the FA genes (VU-SCC-1131, VU-SCC-1365 and OHSU-0974), no correlations were found. Furthermore, the type of *TP53* mutation (wild type, truncating or missense) also showed no correlation with the expression of the nine genes tested. Remarkably, there was a highly significant correlation (Spearman’s rho = 0.95, p<0.001) between *BRCA1* and *BRCA2* expression in the 19 cell lines (Table 1), suggesting that these genes are co-regulated in HNSCC, similar to what has been shown in mammary epithelial cells [35]. The relative mRNA expression of *BRCA1* ranged from 0.31 till 6.5 and for *BRCA2* from 0.20 till 5,0. The expression of *BRCA1* and *BRCA2* also correlated with the expression of *ERCC1*, the gene that is held responsible for cisplatin-DNA adduct repair via the NER pathway.

**Table 1:** Spearman’s rho correlations of the mRNA expression levels of the nine genes examined in 19 HNSCC cell lines.

		CTR1	OCT3	ATP7A	ATP7B	ATM	ATR	BRCA1	BRCA2	ERCC1
CTR1	N		19	19	19	13	19	**19**	*19*	19
	Correlation		−0.026	0.426	0.28	0.487	0.366	**0.586**	*0.534*	0.426
	p		0.915	0.069	0.246	0.091	0.123	**0.008**	*0.019*	0.069
OCT3	N			19	19	13	19	19	19	19
	Correlation			0.214	0.126	−0.187	0.356	−0.034	−0.011	0.282
	p			0.379	0.606	0.541	0.135	0.889	0.966	0.243
ATP7A	N				19	13	*19*	*19*	19	**19**
	Correlation				0.202	0.011	*0.521*	*0.497*	0.398	**0.582**
	p				0.408	0.972	*0.022*	*0.03*	0.091	**0.009**
ATP7B	N					*13*	19	19	19	19
	Correlation					−*0.56*	0.253	0.253	0.298	0.075
	p					*0.046*	0.297	0.296	0.215	0.759
ATM	N						13	13	13	13
	Correlation						0.28	0.033	0.016	0.385
	p						0.354	0.915	0.957	0.194
ATR	N							*19*	19	19
	Correlation							*0.458*	0.367	0.396
	p							*0.048*	0.123	0.093
BRCA1	N								**19**	*19*
	Correlation								**0.953**	*0.494*
	p								**0.000**	*0.032*
BRCA2	N									*19*
	Correlation									*0.517*
	p									*0.023*
ERCC1	N									
	Correlation									
	p									

**Bold** Correlation is significant at the 0.01 level (2-tailed). *Italics* Correlation is significant at the 0.05 level (2-tailed).

### Cisplatin Accumulation and Retention

The cellular cisplatin accumulation was determined after incubating the cells with 75 µM cisplatin during four hours (Table 2). This concentration is not toxic in this short incubation period and allows reliable measurements. The cisplatin accumulation ranged from 91 pmol/10^6^ cells for cell line UM-SCC-14C to 923 pmol/10^6^ cells for cell line UM-SCC-6. Cisplatin retention was determined by incubating the cells with fresh medium during three hours post cisplatin incubation. The maximum efflux was found in cell line VU-SCC-040 (80%, Table 2). There were also three cell lines (UM-SCC-14B, UM-SCC-22A and OHSU-0974) that did not show any cisplatin efflux during the drug-free period.

**Table 2:** Cisplatin accumulation after four hours of cisplatin exposure and the percentage of cisplatin retention after three hours in drug free medium.

HNSCC cell line	IC_50_ (µM)	Cisplatin accumulation (pmol cisplatin/10^6^ cells)	Retention (%)
UM-SCC-6	1.12±0.15	922±56	70.4
UM-SCC-11B	2.48±0.16	153±6	79.6
UM-SCC-14A	3.27±0.51	337±12	69.6
UM-SCC-14B	2.35±0.70	242±62	100
UM-SCC-14C	2.60±0.17	91±13	85.9
UM-SCC-22A	0.69±0.32	97±30	100
UM-SCC-22B	0.93±0.31	452±9	74.2
UM-SCC-38	7.57±1.25	373±22	52.6
VU-SCC-040	1.81±0.45	267±10	20.0
VU-SCC-094	0.91±0.13	ND	ND
VU-SCC-096	0.99±0.46	588±29	56.5
VU-SCC120	4.03±0.92	176±14	87.9
VU-SCC-147	2.97±0.39	ND	ND
VU-SCC-9917	0.99±0.12	258±16	95.5
VU-SCC-OE	1.66±0.25	252±51	98.4
VU-SCC-1131	0.15±0.04	334±114	82.3
VU-SCC-1365	0.80±0.18	275±76	97.3
OHSU-0974	2.99±0.23	275±54	100
FaDu	1.83±0.10	285±90	61.2

Retention (%) represents the remaining cellular cisplatin concentration after three hours in drug free medium. Values are the means of at least duplicate experiments ± SEM. ND; not determined.

Intriguingly, the large range in accumulation did not correlate to the IC_50_ value (p = 0.589, Spearman’s rho = −0.141) nor to any of the mRNA expression levels, among which were the assigned influx pumps CTR1 and OCT3. Elimination of cell lines with a known FA defect from the analysis did not change this observation. The percentage retention also did not show a significant correlation to any of the characteristics of the cell lines, including the mRNA expression of efflux pumps ATP7A and ATP7B. Remarkably, we found a significantly inverse correlation between the cisplatin accumulation and retention (p = 0.015, Spearman’s rho = −0.580), indicating that cells with high cisplatin accumulation also show a strong efflux.

### Cisplatin-DNA Adducts

DNA isolated from cell cultures incubated with 75 µM cisplatin for four hours was subjected to flameless atomic absorption spectrometry (FAAS) in order to determine the level of platinum adducts in the DNA. The resulting data are displayed in Table 3. The platinum content ranged from 0.085 pmol/µg DNA in cell line UM-SCC-14C to 1.235 pmol/ug DNA in VU-SCC-1365 cells.

**Table 3:** Platinum-DNA adducts after four hours of cisplatin exposure and the percentage of adduct retention after three hours in drug free medium.

HNSCC cell line	IC_50_ (µM)	Platinum-DNA adducts (pmol platinum/µg DNA)
UM-SCC-6	1.12±0.15	0.375±0.09
UM-SCC-11B	2.48±0.16	0.525±0.08
UM-SCC-14A	3.27±0.51	0.405±0.13
UM-SCC-14B	2.35±0.70	0.975±0.16
UM-SCC-14C	2.60±0.17	0.085±0.03
UM-SCC-22A	0.69±0.32	0.99±0.00
UM-SCC-22B	0.93±0.31	0.88±0.16
UM-SCC-38	7.57±1.25	0.33±0.06
VU-SCC-040	1.81±0.45	0.48±0.08
VU-SCC-094	0.91±0.13	ND
VU-SCC-096	0.99±0.46	1.12±0.26
VU-SCC120	4.03±0.92	0.27±0.05
VU-SCC-147	2.97±0.39	ND
VU-SCC-9917	0.99±0.12	0.73±0.03
VU-SCC-OE	1.66±0.25	0.395±0.03
VU-SCC-1131	0.15±0.04	0.835±0.09
VU-SCC-1365	0.80±0.18	1.235±0.08
OHSU-0974	2.99±0.23	0.525±0.23
FaDu	1.83±0.10	0.365±0.04

Values are the means of duplicate experiments ± SEM. ND; not determined.

**Figure 3:**
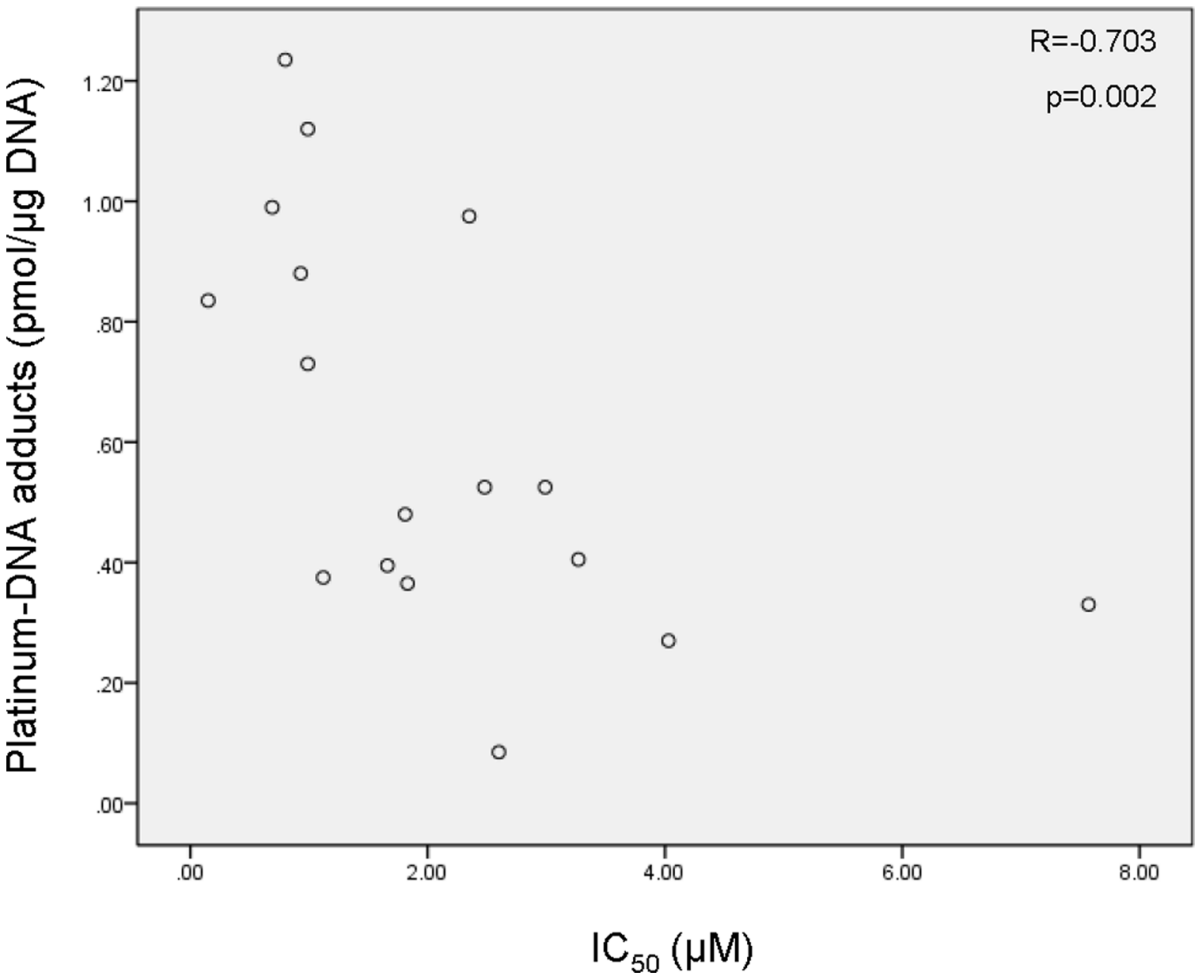
Inverse correlation between IC_50_ value and accumulation of platinum-DNA adducts. The level of DNA-bound platinum was determined in 17 HNSCC cell lines that were incubated with cisplatin. A significant inverse correlation was found between the platinum-DNA adducts and the IC_50_ value. Measurements were performed in duplicate and error bars represent standard deviations.

The most remarkable finding in our study is that the level of cisplatin-DNA adducts showed a significantly inverse correlation with the IC_50_ value (p = 0.002, Spearman’s rho = −0.703), even when we excluded the three cell lines with a known mutation in one of the FA genes (p = 0.006, Spearman’s rho = −0.697). This indicates that cells with a low IC_50_ value for cisplatin contain significantly more DNA adducts compared to cells that are more resistant to cisplatin (Figure 3).

## Discussion

Multiple studies have been performed to find key factors determining the cellular sensitivity to cisplatin. Until now, numerous factors have been identified, but there seems to be no concordance. We evaluated the sensitivity to cisplatin as well as the accumulation and retention of cisplatin and platinum-DNA adducts in a large panel of established HNSCC cell lines with known clinical characteristics. Also the expression levels of several genes known to be involved in cisplatin toxicity were determined. The only parameter that showed a significant correlation to cisplatin sensitivity was the platinum-DNA adduct level. Remarkably, neither the intracellular accumulation nor the retention showed a correlation with either IC_50_ or DNA-bound cisplatin. Apparently, it seems the DNA-bound fraction that determines cisplatin response in head-and-neck cancer more than something else. This suggests that either nuclear transport of cisplatin and/or the level of DNA repair differs between cells, resulting in a different adduct level. It further suggests that assessment of cisplatin-DNA adducts may predict treatment outcome in patients. This has been shown previously but only in small series [36], [37]. At that time the data were obtained by laborious postlabeling techniques to assess adduct levels, but the current highly sensitive FAAS equipment may be much more suited for clinical studies. The remaining major obstacle is the demand for tumor biopsies after cisplatin infusion, which is only possible for easy accessible tumors.

We determined the cisplatin sensitivity in 19 HNSCC cell lines by treating the cells with 18 different cisplatin concentrations during 72 hours. Since pharmacokinetics play an important role in drug availability in patients, it is hard to compare the cisplatin concentrations used in this study to the cisplatin levels that are reached clinically. Patients are nowadays treated with 100 mg/m^2^ cisplatin every three weeks, usually with a one hour intravenous infusion. This administration results in a peak concentration of cisplatin in the blood at a few minutes after stopping the infusion. The peak concentration of free cisplatin has been analyzed and varies between 3.5 and 6 µg/ml [38], which corresponds to approximately 12–18 µM. Furthermore, the total plasma platinum concentration rapidly decreases already within the first two hours after infusion [39]. The cisplatin concentrations used in this study vary from 0.01–666 µM, which corresponds to 3 ng/ml−0.2 mg/ml. This means that we totally cover the range of peak cisplatin concentrations measured for patients. Nonetheless, it is difficult to compare the fixed cisplatin concentration *in vitro* with the rapidly decreasing concentration in patients.

Several studies reported an association between cisplatin sensitivity and *TP53* mutations in solid cancer cell lines *in vitro* [23], [40]–[42], but with conflicting results which might be a consequence of the small sample sizes and different origins of the cell lines tested. However, in the present large panel of HNSCC cell lines we could not find a correlation between the type of *TP53* mutation and the corresponding IC_50_ value. However, our cell line panel contained only two wild type cell lines, and therefore we were unable to determine whether wild type *TP53* significantly altered the sensitivity to cisplatin as compared to cell lines harboring a mutated form of the tumor suppressor gene. Nevertheless, we observed that the two HNSCC cell lines with wild type *TP53* were among the most sensitive cell lines tested. Also *in* *vivo* studies searching for a correlation between *TP53* mutation status and outcome after cisplatin treatment in multiple cancer types led to opposing results [43]–[45]. In case of HNSCC, it is known that approximately 80% of the tumors has an incorrect functioning of the *TP53* pathway by either *TP53* mutation or HPV infection [46], [47], implicating that tumors with wild type *TP53* are rare. This already makes it difficult to imagine that *TP53* mutation status could explain the response to cisplatin.

The influence of copper transporters on the import (*CTR1* [6], [7], [9]) and efflux of cisplatin (*ATP7A* and/or *ATP7B* [11]–[13], [16]) has been observed in a variety of human cancer cell lines. In our data, we could not find a significant correlation between the mRNA expression of *CTR1*, *ATP7A* and *ATP7B* and cisplatin sensitivity. Since the mRNA level does not necessarily reflect the protein level or pump activity within a cell, we might have missed the effect of these genes on the cellular sensitivity to cisplatin. Similarly, we did not find a correlation between *CTR1*, *ATP7A* and *ATP7B* expression and the cisplatin accumulation and retention. On the other hand, mutations that do not influence the expression level of the copper transporters but that do have an effect on the pump activity and the efficiency of cisplatin transport were not detectable with our quantitative real-time PCR approach, although this might have been reflected in an altered cisplatin accumulation, retention or DNA adduct formation. In addition, the subcellular localization of the ATP7A and ATP7B proteins could not be determined in our experimental setup because of very low transcript levels, but might be of importance in the process of cisplatin efflux [48].

Although defects in DNA repair mechanisms are essential for cisplatin induced toxicity, we could not find clear associations between the expression levels of *ERCC1*, *BRCA1* and *BRCA2* and cisplatin sensitivity. Again, this might be a result of the detection method we employed. The *BRCA1* and *BRCA2* genes are described to be often mutated or transcriptionally silenced by promoter methylation, although this has not been observed in HNSCC [49]. Certain polymorphisms in *ERCC1* have been described to be associated with cisplatin sensitivity in multiple types of cancer. These polymorphisms do not alter the expression level of the gene, but might modify the functional activity of the protein.

When comparing the expression levels of the genes tested in our HNSCC panel, we found several significant correlations. Some correlations were explainable when looking at the gene functions. We found a very strong correlation between the expression of *BRCA1* and *BRCA2*, which might point at a possible co-regulation of these genes. It has been shown in prostate cancer and in breast cancer that *BRCA1* and *BRCA2* are indeed co-regulated in response to DNA damage. This phenomenon was also observed in untreated mammary epithelial cells [35]. Furthermore, the expression level of several other genes with distinct functions within a cell (like *ATP7A* and *BRCA1*) showed a significant correlation, but with the current literature at hand no likely explanation for these correlations could be suggested.

From these results we can conclude that the mechanism of cisplatin response seems a relatively simple process in HNSCC. Cells with high IC_50_ values harbor less platinum-DNA adducts. This suggests that the DNA adduct level resulting from cisplatin incubation is the most important determinant of cisplatin sensitivity. Therefore, imaging of radio-labeled cisplatin in patients might be an excellent indicator for treatment outcome. A genome-wide functional siRNA screen might be a useful tool to determine all the genes that specifically determine the cisplatin response and consequently the adduct accumulation level.

## Supporting Information

Table S1 Primer sequences for quantitative real-time PCR.
(DOC)

Table S2 Inhibitory cisplatin concentrations (IC_50_ values) and *TP53* mutation status of 19 HNSCC cell lines.
(DOC)

Table S3 Relative mRNA expression of the indicated genes in 19 HNSCC cell lines.
(XLS)
