# Supplementary material for: DNA-Bound Platinum Is the Major Determinant of Cisplatin Sensitivity in Head and Neck Squamous Carcinoma Cells
Source: PLoS One. 2013 Apr 17;8(4):e61555. doi: 10.1371/journal.pone.0061555 (PMC3629194; doi:10.1371/journal.pone.0061555)
Supplement: Table S1 — Primer sequences for quantitative real-time PCR. (DOC) [file pone.0061555.s001.doc]

**Supporting Table S1. Primer sequences for quantitative real-time PCR.**

| **Gene** | **Forward primer sequence (5’-3’)** | **Reverse primer sequence (5’-3’)** |
| --- | --- | --- |
| *CTR1* | TGACGGGTTAAGATTCGGAG | TGGTGGGAATGATCCATTTT |
| *OCT1* | TCTTCCATCGTCACTGAGTTCAAC | AGAAGCCCGCATTCAAACAG |
| *OCT2* | CGGAGATATCGGAGAACAGT | GATCTCCCAGAATAAGAATGC |
| *OCT3* | GCTTCCTGACTGGAGCATTC | AACACCAAGGCAGGATAGCA |
| *ATP7A* | TGGCTGCTTCATCTGTTTCTGT | CGGGCAGGCAGTTCATAACT |
| *ATP7B* | CTCAGAAGCCCTGGCTAAA | CTCCATGGGGACTTGCTC |
| *ATM* | GGCATTCAGATTCCAAACAA | GGCTGATACATTTGGTTTTGC |
| *ATR* | AGTGACCATCTATCTTCTTCCAC | TTCTGCATAAACCTCCTGCT |
| *BRCA1* | CCCAGAAGAATTTATGCTCGT | ACAACATGAGTAGTCTCTTCAG |
| *BRCA2* | CCTGTCCACTTCTAAATTCTTGTC | CCACATACCACTGACTTATCTC |
| *ERCC1* | TGCTTGTCCAGGTGGATGTG | TGTGCAGTCGGCCAGGATA |
| *GUSB* | GAAAATATGTGGTTGGAGAGCTCATT | CCGAGTGAAGATCCCCTTTTTA |
