# Supplementary material for: DNA-Bound Platinum Is the Major Determinant of Cisplatin Sensitivity in Head and Neck Squamous Carcinoma Cells
Source: PLoS One. 2013 Apr 17;8(4):e61555. doi: 10.1371/journal.pone.0061555 (PMC3629194; doi:10.1371/journal.pone.0061555)
Supplement: Table S2 — Inhibitory cisplatin concentrations (IC50 values) and TP53 mutation status of 19 HNSCC cell lines. (DOC) [file pone.0061555.s002.doc]

**Supporting Table S2. Inhibitory cisplatin concentrations (IC_50_ values) and *TP53* mutation status of 19 HNSCC cell lines.**

| **HNSCC cell line** | **Primary tumor site** | **Stage** | **IC_50_ (µM)** | ***TP53* mutation type** | **Genomic description^§^** | **Protein description^§^** | **Mutation effect** | ***TP53* mutation classification[28]** |
| --- | --- | --- | --- | --- | --- | --- | --- | --- |
| UM-SCC-6 | Base of tongue | T2N0 | 1.12 ± 0.15 | Wild-type |  |  |  |  |
| UM-SCC-11B | Larynx | T2N2a | 2.48 ± 0.16 | Point mutation | g.14052G>C | p.C242S | Missense | Missense |
| UM-SCC-14A | Floor of mouth | T1N0 | 3.27 ± 0.51 | Deletion | g.14499_14528del | p.C277_E287delinsX | Stop codon | Truncating |
|  |  |  |  | Point mutation | g.14509A>T | p.R280S | Missense | Missense |
| UM-SCC-14B^a)^ | Floor of mouth | T1N0 | 2.35 ± 0.70 | Deletion | g.14499_14528del | p.C277_E287delinsX | Stop codon | Truncating |
|  |  |  |  | Point mutation | g.14509A>T | p.R280S | Missense | Missense |
| UM-SCC-14C^b)^ | Floor of mouth | T1N0 | 2.60 ± 0.17 | Deletion | g.14499_14528del | p.C277_E287delinsX | Stop codon | Truncating |
|  |  |  |  | Point mutation | g.14509A>T | p.R280S | Missense | Missense |
| UM-SCC-22A | Hypopharynx | T2N1 | 0.69 ± 0.32 | Point mutation | g.13419A>G | p.Y220C | Missense | Missense |
|  |  |  |  | Point mutation | g.14754+1G>T | NA | Splice | Truncating |
| UM-SCC-22B^c)^ | Hypopharynx | T2N1 | 0.93 ± 0.31 | Point mutation | g.13419A>G | p.Y220C | Missense | Missense |
|  |  |  |  | Point mutation | g.14754+1G>T | NA | Splice | Truncating |
| UM-SCC-38 | Tonsillar pillar | T2N2a | 7.57 ± 1.25 | Point mutation | g.13075G>T | p.K132N | Missense | Missense |
| VU-SCC-040 | Tongue | T3N0 | 1.81 ± 0.45 | Wild-type |  |  |  |  |
| VU-SCC-094 | Tongue | T3N1 | 0.91 ± 0.13 | Deletion | g.14588+1G>A | NA | Splice | Truncating |
| VU-SCC-096 | Trigonum retromolare | T4N1 | 0.99 ± 0.46 | Point mutation | g.13338 A>T | p.H193L | Missense | Missense |
| VU-SCC-120 | Tongue | T3N1 | 4.03 ± 0.92 | Mutation multiple bases | g.13160/13161GC>TT | p.A161F | Missense | Missense |
|  |  |  |  | Point mutation | g.13206G>A | p.C176Y | Missense | Missense |
| VU-SCC-147 | Floor of mouth | T4N2 | 2.97 ± 0.39 | Point mutation | g.14097T>G | p.L257R | Missense | Missense |
| VU-SCC-9917 | Oral cavity | T2N2b | 0.99 ± 0.12 | Point mutation | g.13346C>T | p.R196X | Stop codon | Truncating |
| VU-SCC-OE | Floor of mouth | NA | 1.66 ± 0.25 | Deletion | g.11727_14754del | p.M1_Q331del | Nonsense | Truncating |
| VU-SCC-1131 | Floor of mouth | T4N2b | 0.15 ± 0.04 | Point mutation | g.14487G>T | p.R273L | Missense | Missense |
| VU-SCC-1365 | Mouth mucosa | NA | 0.80 ± 0.18 | Point mutation | g.14513C>T | p.R282W | Missense | Missense |
| OHSU-0974 | Tongue | NA | 2.99 ± 0.23 | Deletion | g.12033delT | p.S37fs | Frameshift | Truncating |
| FaDu | Hypopharynx | NA | 1.83 ± 0.10 | Point mutation | g.14070G>T | p.R248L | Missense | Missense |

^a)^ Local recurrence of UM-SCC-14A ^b)^ Local recurrence of UM-SCC-14A ^c)^ Metastasis of UM-SCC-22A

**^§^** IARC TP53 Database Download TP53Somatic R12 Genbank: X54156

NA; not annotated
